# Ejaculation Effect on Canine Prostatic Specific Esterase Serum Concentration

**DOI:** 10.3390/ani10030381

**Published:** 2020-02-27

**Authors:** Salvatore Alonge, Monica Melandri, Raffaella Leoci, Giovanni M. Lacalandra, Michele Caira, Giulio G. Aiudi

**Affiliations:** 1 Società Veterinaria “Il Melograno” Srl, 21018 Sesto Calende, Italy; drmonicamelandri@gmail.com; 2 Department of Veterinary Medicine (DiMeV), Section of Clinical Obstetrics, University of Bari “Aldo Moro”, 70010 Valenzano, Italy; leocivet@yahoo.it (R.L.); giovannimichele.lacalandra@uniba.it (G.M.L.); michele.caira@uniba.it (M.C.); giulioguido.aiudi@uniba.it (G.G.A.)

**Keywords:** CPSE, dog, ejaculation, sexual rest

## Abstract

**Simple Summary:**

Canine andrology has become an important area within veterinary practice. In this field, the prostate plays a crucial role; it can be affected by several illnesses, strongly influencing male fertility. The diagnosis of such diseases relies on different procedures; among them, following the example of human medicine, the study of serum biomarkers has led to the use of Canine Prostatic Specific Esterase (CPSE) as a first-line tool. The CPSE is influenced neither by circadian rhythms nor by transrectal palpation. The present study aimed to evaluate the effect of ejaculation on CPSE, both in healthy animals and in subjects with prostatic disorders. Soon after ejaculation, CPSE concentrations in serum are significantly higher than basal ones; they then return to their original concentrations in 24 h. At all times, CPSE is higher in affected dogs than in normal subjects; however, some healthy patients could be misdiagnosed with prostatic disorders based on the CPSE concentrations measured soon after ejaculation. Thus, in accordance with recent reports on canine prostate ultrasonography, a sexual rest of minimum 24 h should be applied before a thorough examination of the male genital tract.

**Abstract:**

Canine prostatic diseases are usually asymptomatic in their onset and often identified in advanced stages. Canine prostatic specific esterase (CPSE) represents an early serum marker for prostatic diseases, also in asymptomatic dogs. The present study aimed to identify the effects of ejaculation on serum CPSE. Twenty adult intact male dogs were enrolled. Blood samples were collected to measure CPSE concentrations before (T0), immediately after (T1), and 24 h post (T2) ejaculation. Data were compared within and between groups by ANOVA (*p* < 0.05). Dogs were divided in two equal groups: A (healthy: CPSE ≤ 52.3 ng/mL at T0) and B (suspected for prostatic disorders: CPSE > 52.3 ng/mL or diagnosed with symptoms of prostatic diseases: CPSE > 90 ng/mL). CPSE was shown to be statistically higher in group B than A at any time point. In both groups, CPSE showed a significant increase at T1, and no significant differences between T0 and T2. This study demonstrates a definite effect of ejaculation on CPSE concentration. Twenty-four hours post-ejaculation, CPSE returns to basal values. Such physiological effects of ejaculation should be considered when planning analyses of CPSE concentrations, i.e., by respecting a proper sexual rest.

## 1. Introduction

Canine prostatic diseases, mainly represented by benign prostatic hyperplasia (BPH), prostatic cysts, prostatitis, and neoplasias, are usually asymptomatic at their onset, and thus, are often identified in advanced stages, in studs as well as in pets [1]. A screening programme for prostatic diseases could make it possible to achieve a more accurate evaluation of the real prevalence of prostatic pathologies together with earlier diagnoses. This could be easily achieved by blood samples, based on the availability of serum biomarkers [2,3]. In the search for earlier diagnostic timings and new therapeutic strategies, several researchers have evaluated canine serum biomarkers, similarly to biomarkers used in humans on a routine basis [2,3,4,5,6]. Alkaline phosphatase, carnitine, and Canine Prostatic Specific Esterase (CPSE) are the three most common biomarkers used to evaluate the male canine reproductive tract [7]. In dogs, alkaline phosphatase and carnitine are not specific for the prostate gland, as they can be brought back to disorders of the epididymis and ductal network [7]. Serum prostatic acid phosphatase and, mainly, Prostate Specific Antigen (PSA) are successfully used to diagnose prostatic carcinoma in men, but the role of such biomarkers is still debated in dogs. The first yielded inconclusive results, while the latter did not seem to peak in canine prostatic neoplasias [8,9,10], even if some recent studies report an elevation of PSA along with experimentally induced BPH [11,12]. Recently, the literature has reported higher CPSE concentrations in dogs affected by several prostatic conditions, represented by BPH, bacterial prostatitis, and prostatic carcinoma [5,13,14,15,16,17]. Since then, CPSE has been extensively used in the diagnosis of prostatic disorders on a routine basis, as a marker for diseases of the gland, while additional second and third level analyses are required to identify the specific pathologies affecting the prostate [2,5,6]. It was recently suggested that dogs showing clinical symptoms have an increased prostatic volume, i.e., higher than 2.5 times the normal expected one, and a CPSE concentration of over 90 ng/mL [2]. Moreover, CPSE, the main prostatic secretory product, can represent an early quality serum marker for prostatic diseases, also in asymptomatic patients [3]. A threshold of 52.3 ng/mL for serum CPSE was suggested as a mean to recognize dogs requiring further accurate evaluations, since such values are commonly associated with echographic changes and increased prostate dimensions, even in subjects that are still asymptomatic [3,6].

Thus, since CPSE measurement can play a crucial role in the management of prostatic health in dogs, any condition that can cause its elevation has to be identified. Besides defining specific features of CPSE due to peculiar prostatic disorders or links to different stages and severities of prostatic conditions, clinicians should become aware of the possible existence of physiological fluctuations in the concentrations of CPSE. In light of this, it was recently demonstrated that neither circadian rhythms nor transrectal palpation affect CPSE concentrations [18].

To examine this issue further, the present prospective clinical study was designed to investigate the effect of ejaculation on serum CPSE concentrations in dogs, and to determine how long this effect lasts.

## 2. Materials and Methods

### 2.1. Ethics

This study was performed in accordance with the ethical guidelines of the animal welfare committee. Institutional Review Board approval of the study was obtained by the University of Bari “Aldo Moro,” Ethic Committee DETO, Italy (Protocol N 35/17 DETO; 26 June 2017). Procedures with animals were performed following good veterinary practice for animal welfare according to national laws in force (D.Lgs 116/92).

Informed owner consent was obtained.

### 2.2. Animals

Patients were enrolled as volunteers among dogs registered in the database of the University Hospital by phone contact.

The goal was to include 10 adult intact male dogs that were negative in a CPSE screening test (group A) and 10 adult intact male dogs that were positive (group B), with the latter subdivided as borderline (*n* = 5 dogs) or positive (*n* = 5 dogs).

One week before the day selected for the procedure, each dog underwent a clinical examination including a thorough history, a rectal exploration of the prostate, and an ultrasonographic evaluation of the gland. At the same time, the positive reaction of all enrolled subjects to semen collection by manual stimulation was verified, exploiting, if necessary, swabs of females in oestrus [19]. Sexual rest was then required for the whole week between this test and the day of the procedure to avoid any interference effects.

The following specific clinical signs of prostatic disorders were considered: preputial sero-sanguineous discharge; difficulties in defecation; pelvic pain (sometimes expressed as lameness and/or pain upon abdominal palpation or rectal exploration), recurrent urinary tract diseases (i.e., urinary tract infections); infertility, reduced fertility, or spermiogram alterations.

The ultrasonography of the prostate evaluated alterations of the following aspects: volume (expressed as V-ratio [2]), borders, position, echotexture, and ultrastructure (i.e., alteration of echogenicity), presence of cysts and/or other neoformations.

Apart from potential prostatic disorders, all dogs were healthy and not receiving any drugs or therapy.

### 2.3. Procedure

In each dog, three venous blood samples were collected from the cephalic vein into an empty plastic vial to measure serum CPSE. Venous blood samples were collected before (T0, basal value) and immediately after (T1) ejaculation (at the end of the collection of the third ejaculatory fraction), and 24 h later (T2). Blood samples were immediately centrifuged, serum samples were separated, moved to empty Eppendorf vials, and stored at −20 °C until CPSE was analysed using a commercial assay, consisting of a quantitative immunocromatographic test which was able to detect serum CPSE concentration by a laser-induced fluorescence analysis (Speed Reader, Virbac, Milan, Italy).

Animals were enrolled in the study, when, according to the results obtained at the breeding soundness examination and at the T0 (basal value) CPSE dosage, it was possible to assign them to one of the two study groups. Table 1 reports data concerning the 10 subjects included in group A (healthy dogs: T0 serum CPSE ≤ 52.3 ng/mL, without any clinical nor echographic or clinical signs of prostatic disorders) and of the 10 gathered in group B (B1: animals suspected for prostatic disorders: T0 serum CPSE > 52.3 ng/mL, without any evident clinical signs of prostatic disorders, but with already one or more detectable ultrasonographic alterations, and a V-ratio > 1.5; and B2: diagnosed with clinical signs of prostatic diseases: T0 serum CPSE > 90 ng/mL, with one or more clinical signs and one or more echographic alterations which was attributable to a prostatic disorder, and a V-ratio > 2.5) [2,3].

### 2.4. Statistical Analysis

All CPSE concentrations obtained were compiled in Excel 2010 Office files. Mean values ± SD were calculated for each parameter. The normality of data distribution was checked by a Shapiro-Wilk test. Data were statistically compared first within each group upon each sampling time by repeated ANOVA measures to evaluate the possible effect of ejaculation on CPSE concentration and its persistence over time. Then data were statistically compared between groups on each sampling time by a *t*-test, to verify whether CPSE concentrations always remained higher in group B than in group A. Age and bodyweight were compared between groups by *t*-test. Statistical significance was considered with *p* ≤ 0.05. A statistical analysis was performed with the online tool VassarStats: Website for Statistical Computation (http://vassarstats.net, Vassar College, New York, NY, USA).

## 3. Results

No statistically significant differences were reported concerning bodyweight between the two study groups (24.75 ± 9.87 kg in group A vs. 30.03 ± 9.55 kg in group B; *p* ≥ 0.05).

In group A, age was significantly lower than in group B (2.2 ± 0.71 years in group A vs. 3.4 ± 0.81 years in group B; *p* ≤ 0.05).

In both groups, CPSE showed a statistically significant increase at T1 (T0 vs. T1, *p* ≤ 0.05), while there was no difference between T0 and T2 (T0 vs. T2, *p* ≥ 0.05), as reported in Table 2.

The mean increase of CPSE concentration from T0 to T1 was 56% ± 49 in group A and 56% ± 52 in group B.

At T2, 90% of patients of each group came back to CPSE basal values, allowing to classify them as at T0 [3]. At T2, patient ID7 (T0 CPSE 51.68 ng/mL) still had a CPSE concentration of 62.2 ng/mL, leading to the erroneous classification into group B1 group instead of the A group, while subject ID13 (T0 CPSE 75.52 ng/mL) reported a CPSE of 98.26 ng/mL, leading to a possible mistake in the subject’s attribution to group B2 instead of B1.

At each sampling time, CPSE levels were statistically higher (*p* ≤ 0.05) in group B (T0: 123.79 ± 74.42 ng/mL; T1: 197.06 ± 135.51 ng/mL; T2: 104.26 ± 38.64 ng/mL) than in group A (T0: 36.92 ± 13.33 ng/mL; T1: 55.07 ± 22.08 ng/mL; T2: 36.12 ± 16.19 ng/mL), as reported in Table 2.

## 4. Discussion

CPSE has been identified as a suitable biomarker to be included in a prostate health screening programme in dogs, which should be periodically performed in canine patients following the procedures of human medicine related to regularly timed screenings for prostatic health, mainly based on PSA [6,14]. The clinical usefulness of any biomarker depends on the knowledge about its physiological features. In order to increase the specificity of the CPSE test, factors possibly affecting its concentration, apart from prostatic disorders, should be disclosed. This study demonstrated a definite effect of ejaculation on CPSE concentration. Respecting sexual rest for 24 h before CPSE analysis is crucial to avoid possible overdiagnoses of BPH-related disorders in healthy patients. In fact, serum CPSE rises at T1, when patients from group A showed mean CPSE values above the threshold, that could have led, in some cases, to a false positive diagnosis [2,3].

Also in human medicine, ejaculation has been claimed to be one of the factors affecting PSA values, and its effect has been evaluated in several studies, reporting at first conflicting results with increased, unchanged, or decreased PSA concentrations after ejaculation [20,21,22,23,24,25,26,27,28,29,30,31,32,33]. Nevertheless, in works reporting decreased or unchanged PSA concentrations, sampling time intervals after ejaculation were too long (1 to 7 days after ejaculation) to detect early PSA elevations, which are instead observed during the very first hours [21]. Recently, it was definitively stated that the serum concentration of PSA increases at 1 h after ejaculation and returns to baseline values at 24 h [20,21,29] in 92% of patients [27]. The present study demonstrated that 24 h after ejaculation, increased serum CPSE came back to basal values in 90% of tested animals, following a trend similar to that reported for PSA in human medicine [20,21,27,29]. The relationship between basal and post-ejaculation CPSE is the same in negative dogs (group A), and in positive animals, i.e., those either suspected to have or diagnosed with glandular BPH-related diseases (group B); no statistically significant differences were found in CPSE mean concentrations for both groups between T0 and T2.

In dogs, it was recently reported that ejaculation induces an increase in the vascularisation of the prostate gland [34,35,36,37]. Augmented prostatic vascular flow can be detected up to 24 h after ejaculation by Power Doppler [34], which is very accurate for slow flows [36]. On the other hand, Pulsed-Wave Doppler, more specific for high flows, remains altered for a period of minimum 6 h after ejaculation [35]. It can be inferred that the strongly increased blood supply transports a greater CPSE amount away from the gland, inducing an increase at T1. Progressively, the vascularisation returns to its original conditions and serum CPSE comes back to basal values in approximately 24 h.

In fact, serum biomarker concentrations are dependent on the production of the biomarker itself, and its passage into the bloodstream [6,20]. Normal prostatic epithelium is constituted of PSA- (in men) and CPSE (in dogs)-secreting acinar tissue. A basement membrane and a cellular layer separate these cells from lymphatic vessels and capillaries. Then, PSA and CPSE are transported through ejaculatory ducts to the urethra to reach and constitute seminal plasma [6,20]. It can be hypothesized that, as reported in human medicine regarding PSA [20], during its transportation, CPSE can enter into the systemic circulation and its increase might be due to a CPSE wash-back into the bloodstream. Particularly, while pelvic muscles and peri-prostatic tissue contractions of ejaculation may increase the leakage of PSA [31] and CPSE into the bloodstream, the increased vascular bed revealed at the ultrasonographic examination [34,35] drives the biomarkers to the systemic circulation.

The present study showed that the higher basal concentrations of CPSE are, the higher the post-ejaculatory peak; they are both more pronounced in subjects affected by prostatic disorders. In human medicine, too, the rise of PSA concentrations after ejaculation is more pronounced in men with larger prostate volumes and higher original PSA [21]. A hypothesis explaining higher PSA and CPSE concentrations in elderly patients states that the described physiologic barrier can weaken, becoming more permeable, resulting in the passage of higher amounts of PSA [21] and CPSE into the bloodstream. Moreover, in subjects with BPH-related disorders, ductal obstruction, acinar dilatation, and secretion retention might increase PSA [20,21] and CPSE leakage during ejaculation.

The change in serum PSA after ejaculation does not depend on patient age [19]. The same trend was demonstrated for CPSE in the present study; even if age was significantly higher in group B, the trend in CPSE concentration oscillations was not different between the two study groups, as shown by the *t*-test analysis performed at T0, T1, and T2. A similar trend for CPSE was observed in the present study: patients came back to CPSE basal values 24 h after ejaculation, independent of age and study group.

In human medicine [27,28], it was reported that PSA can be altered and increased by several procedures, including prostate manipulation or massage [37], digital rectal examination [38], prostate needle biopsy [38], cystoscopy [39], and transurethral resection of the prostate [39]. Serum PSA can also be affected by extraprostatic disorders, such as acute urinary retention [39], but never shows diurnal variations [22].

In dogs, transrectal palpation of the prostate was reported not to affect CPSE concentrations in serum [18]. Up to now, no studies have been conducted to evaluate the effect of more invasive prostatic procedures on CPSE serum concentrations, as these operations are limited to exceptional singular cases in veterinary medicine [10], or to consider the relevance of coexisting diseases. Also, CPSE has been proven not to be affected by circadian rhythm [18]. Moreover, a study from human medicine highlighted that hospitalization may induce a relevant decrease in serum PSA 24 h after hospital admission [39]. Further studies would be advisable to verify if the same decrease takes place also in dogs. However, in the present study, to avoid this possible bias, patients were not hospitalized during the procedures.

A possible limiting factor of this study is the recruiting system of subjects. Sampling was not casual, and dogs were voluntarily selected, according to specific features of their prostatic conditions, to include healthy as well as borderline and diseased patients. The aim of the study was to highlight the possible effect of ejaculation on serum CPSE concentrations, independent of the prostatic conditions. As a consequence, such an effect should have been verified in dogs showing all possible clinical features, with negative, borderline, and positive CPSE basal values, in order to equally represent subjects from any possible epidemiological condition in the canine population which can be encountered in veterinary clinical practice. This system of enrolment is probably responsible for the statistically significant difference in terms of age reported between the two study groups. As prostatic disorders are more common in elderly patients, young animals were expressly sought out for inclusion in group A; thus, the present results show a higher prevalence of positive CPSE screening tests in older dogs (group B).

## 5. Conclusions

The efficacy of screening and early detection of prostatic disorders in dogs has been proven, and CPSE is an established marker for the diagnosis and monitoring of prostatic disorders. Identifying factors that spuriously affect CPSE concentrations would make it possible to optimize the accuracy of the screening and of the early detection of prostatic diseases, increasing diagnostic success, and lowering false-positive rates for CPSE dosages. The physiological effects of ejaculation highlighted by the present study should be taken into account whenever CPSE dosage is planned either to select patients needing further prostatic evaluations or to reach a definitive diagnosis.

A proper sexual rest of minimum 24 h is recommended before the examination to exploit this diagnostic tool at best. In controlling known physiological factors, such as ejaculation, which possibly affect serum CPSE, this biomarker could become even more reliable. Moreover, especially referring to an asymptomatic population, false-positive CPSE test results could be avoided, limiting a high number of unnecessary additional exams and procedures.

## Figures and Tables

**Table 1:** Patients enrolled in the study.

Patient ID	Breed	Body Weight (kg)	Age (Years)	Study Group
1	Weimaraner	29	1.5	A
2	Pointer	20	1.5	A
3	Dachshund	5	1.5	A
4	Weimaraner	34	2	A
5	Weimaraner	31.5	3	A
6	Jack Russell Terrier	13	3	A
7	Weimaraner	34	2	A
8	Weimaraner	31.5	3.5	A
9	Pointer	20	2	A
10	Weimaraner	29.5	2	A
11	French Bulldog	15	3	B1
12	Labrador Retriever	32	2.5	B1
13	Labrador Retriever	34	2.5	B1
14	French Bulldog	12.8	3.5	B1
15	Chow-Chow	30	2.5	B1
16	Cane Corso	37	5	B2
17	Siberian Husky	25.5	3	B2
18	Rottweiler	39.5	3.5	B2
19	Rottweiler	40	4	B2
20	Bloodhound	34.5	4	B2

**Table 2:** CPSE concentrations (ng/mL) in Group A and Group B dogs, expressed as mean ± SD.

Group/Time	T0	T1	T2
Group A	36.92 ± 13.33^a^	55.07 ± 22.08^b^	36.12 ± 16.19^a^
Group B	123.79 ± 74.42^c^	197.06 ± 135.51^d^	104.26 ± 38.64^c^

Different superscripts denote statistically significant differences within rows and columns (repeated measures ANOVA and *t*-test, respectively; *p* ≤ 0.05).
